# Oxidative Stress Markers Induced by Hyperosmolarity in Primary Human Corneal Epithelial Cells

**DOI:** 10.1371/journal.pone.0126561

**Published:** 2015-05-29

**Authors:** Ruzhi Deng, Xia Hua, Jin Li, Wei Chi, Zongduan Zhang, Fan Lu, Lili Zhang, Stephen C. Pflugfelder, De-Quan Li

**Affiliations:** 1 School of Optometry and Ophthalmology, Wenzhou Medical University, Wenzhou, China; 2 Ocular Surface Center, Cullen Eye Institute, Department of Ophthalmology, Baylor College of Medicine, Houston, Texas, United States of America; 3 Tianjin Eye Hospital, Tianjin Key Lab of Ophthalmology and Visual Science, Clinical College of Ophthalmology, Tianjin Medical University, Tianjin, China; Indian Institute of Toxicology Reserach, INDIA

## Abstract

Oxidative stress has been known to be involved in pathogenesis of dry eye disease. However, few studies have comprehensively investigated the relationship between hyperosmolarity and oxidative damage in human ocular surface. This study was to explore whether and how hyperosmolarity induces oxidative stress markers in primary human corneal epithelial cells (HCECs). Primary HCECs were established from donor limbal explants. The hyperosmolarity model was made in HCECs cultured in isosmolar (312 mOsM) or hyperosmotic (350, 400, 450 mOsM) media. Production of reactive oxygen species (ROS), oxidative damage markers, oxygenases and anti-oxidative enzymes were analyzed by DCFDA kit, RT-qPCR, immunofluorescent and immunohistochemical staining and Western blotting. Compared to isosmolar medium, ROS production significantly increased at time- and osmolarity-dependent manner in HCECs exposed to media with increasing osmolarities (350–450 mOsM). Hyperosmolarity significantly induced oxidative damage markers in cell membrane with increased toxic products of lipid peroxidation, 4–hydroxynonenal (4-HNE) and malondialdehyde (MDA), and in nuclear and mitochondria DNA with increased aconitase-2 and 8-OHdG. Hyperosmotic stress also increased the mRNA expression and protein production of heme oxygenase-1 (HMOX1) and cyclooxygenase-2 (COX2), but reduced the levels of antioxidant enzymes, superoxide dismutase-1 (SOD1), and glutathione peroxidase-1 (GPX1). In conclusion, our comprehensive findings demonstrate that hyperosmolarity induces oxidative stress in HCECs by stimulating ROS production and disrupting the balance of oxygenases and antioxidant enzymes, which in turn cause cell damage with increased oxidative markers in membrane lipid peroxidation and mitochondrial DNA damage.

## Introduction

Reactive oxygen species (ROS) which are chemically reactive molecules containing oxygen are formed as a natural byproduct of the normal metabolism of oxygen and have important roles in cell signaling and homeostasis [1]. However, high levels of ROS cause oxidative stress and cell injury, including at least three reactions, lipid peroxidation of membranes, intracellular oxidative modification of proteins, and oxidative damage to DNA [2–5]. These oxidative reactions form adducts with lipids, protein and DNA, and lead to decreased functions of intracellular organelles and further damage [6,7]. Excessive ROS cause**s** oxidative stress, which reflects an imbalance between the systemic manifestation of ROS and a biological system's ability to readily detoxify the reactive intermediates or to repair the resulting damage. Oxidative stress excites oxidative damage and has been involved in pathogenesis of many disease, such as cancer, Parkinson's disease, Alzheimer's disease, heart failure, lung and skin diseases [8–11]. Oxidative stress has also been reported to play an important role in ocular diseases including ocular surface inflammation and dry eye disease [12–14].

Dry eye affects 14% to 33% of adult population worldwide. Dry eye is often accompanied by increased tear osmolarity and ocular surface inflammation. Reduced aqueous tear flow and/or increased evaporation of the aqueous tear phase often lead to tear hyperosmolarity, a key step in the vicious circle of dry eye disease pathology in ocular surface epithelium [15]. Hyperosmolarity has been considered as a key factor that initiates the ocular surface inflammation and apoptosis in dry eye patients, dry eye mouse models, as well as in vitro hyperosmotic culture models of human corneal epithelial cells (HCECs) [15–20].

Recent studies have demonstrated that oxidative stress is involved in pathogenesis of dry eye disease. Higher levels of ROS, lipid oxidative stress markers and inflammatory cells were found in the conjunctiva and tear film of Sjögren syndrome patients [21] and different dry eye animal models [13,14,22,23]. However, few studies have comprehensively investigated the relationship between hyperosmolarity and oxidative damage in human ocular surface. The present study was to explore whether and how hyperosmolarity induces oxidative injury to ocular surface epithelium using an in vitro primary HCEC culture model.

## Materials and Methods

### Materials and reagents

Cell culture dishes, plates, centrifuge tubes, and other plastic ware were purchased from BD Biosciences (Lincoln Park, NJ); Dulbecco modified Eagle medium (DMEM), Ham F-12, amphotericin B, and gentamicin were from Invitrogen (Grand Island, NY). Fetal bovine serum (FBS) was from Hyclone (Logan, UT). RNeasy Plus Mini RNA extraction kit from Qiagen (Valencia, CA); Ready-To-Go-Primer First-Strand Beads were from GE Healthcare (Piscataway, NJ); TaqMan gene expression assays and real-time PCR master mix were from Applied Biosystems (Foster City, CA). DCFDA—Cellular Reactive Oxygen Species Detection Assay Kit, rabbit polyclonal antibody against human malondialdehyde (MDA), 8-hydroxy-2-deoxyguanosine (8-OHdG), 4-hydroxy-2-nonenal (HNE), aconitase-2, glutathione peroxidase-1 (GPX1), and mouse monoclonal antibody against superoxide dismutase-1 (SOD1) were purchased from Abcam (Cambridge, MA). Rabbit polyclonal antibody against human heme oxygenase-1 (HMOX1) and cyclooxygenase-2 (COX2) were from Santa Cruz Biotechnology (Santa Cruz, CA). β-actin antibody was from BioLegend (San Diego, CA). Fluorescein Alexa-Flour 488-conjugated secondary antibodies (donkey anti-rabbit, or goat anti-mouse IgG) were from Molecular Probes (Eugene, OR).

### Primary cultures of HCECs and in vitro hyperosmolarity model

Human donor corneoscleral tissues (<72 h after death) not suitable for clinical use, from donors aged 19 to 67 years, were obtained from the Lions Eye Bank of Texas (Houston, TX). Primary HCECs were cultured in 12-well plates using explants from corneal limbal rims in a supplemented hormonal epidermal medium (SHEM) containing 5% FBS using our previous methods [24]. Confluent primary corneal epithelial cultures in 14–18 days were switched to an equal volume (0.5 mL/well) of serum-free medium (SHEM without FBS) overnight, and then treated for 4 or 24 h with isosmotic (312 mOsM) or hyperosmolar media (350, 400 or 450 mOsM), which were achieved by adding (19, 44 or 69 mM) sodium chloride (NaCl). The osmolarity of the culture media was measured by a vapour pressure osmometer in the Body Fluid Chemistry Clinical Laboratory of the Methodist Hospital (Houston, TX). The cells treated for 4 h were lysed in RLT buffer from Qiagen RNeasy Plus Mini kit, and subjected to RNA extraction for gene expression assay. The cells treated for 24–48 h were used for protein assays such as Western blot, immunofluorescent or immunohistochemical staining. Each experiment was repeated 3–5 times, and data were representatively and/or statistically presented in the Results with figures.

### RNA extraction, reverse transcription, and quantitative real-time PCR (RT-qPCR)

Total RNA was extracted with a RNeasy Plus Mini Kit (Qiagen, Valencia, CA) according to the manufacturer’s instructions, quantified with a spectrophotometer (NanoDrop ND-1000; Thermo Scientific, Wilmington, DE), and stored at -80°C before use. The first strand cDNA was synthesized by RT from 1.0 μg of total RNA using Ready-To-Go You-Prime First-Strand Beads as previously described [19,25]. Quantitative real-time PCR was performed in a Mx3005P QPCR System (Stratagene, La Jolla, CA) with 20 μl reaction volume containing 5 μl of cDNA, 1 μl gene expression assay and 10 μl gene expression master mix (TaqMan; ABI). TaqMan gene expression assays used for this study were: GAPDH (Hs99999905_m1), HMOX1 (Hs01110250_m1) and COX2 (Hs00153133_m1). The thermocycler parameters were 50°C for 2 min and 95°C for 10 min, followed by 40 cycles of 95°C for 15 s and 60°C for 1 min. A non-template control was included to evaluate DNA contamination. The results were analyzed by the comparative threshold cycle (Ct) method and normalized by GAPDH as an internal control [26].

### Measurement of cellular ROS production

Cellular ROS production was measured using a DCFDA assay kit according to manufactory’s protocol. DCFDA (2’,7’-dichlorofluorescein diacetate), a cell-permeable fluorogenic dye, is deacetylated by cellular esterases to a non-fluorescent compound, and later oxidized by ROS into highly fluorescent 2’,7’-dichlorofluorescein (DCF), which measures hydroxyl, peroxyl and other ROS activity within the cell. HCECs were grown on 96-well plates or 8-chamber slides. When reached confluence, the cells were washed twice with phosphate-buffered saline (PBS) and then incubated with 25 μM DCFDA in essential medium with 10% FBS in 37°C incubator for 45 min. After PBS washed twice, the cells were exposed to hyperosmotic media (350, 400 or 450 mOsM) by adding NaCl (19, 44 or 69 mM, respectively) for different time periods (30–180 min). Cells images were taken under fluorescent microscope. Cell fluorescence in 96-well plates was measured at 488 nm excitation and 525 nm emission using Tecan Infinite M200 Multimode Microplate Reader (Tecan US, Inc. Morrisville, NC) after adding NaCl for 15–120 mins. Relative changes of DCF fluorescence were expressed as fold increase over untreated cells.

### Immunofluorescent and immunohistochemistry staining

The human corneal epithelial cells on 8-chamber slides were fixed with freshly prepared 2% paraformaldehyde at 4°C for 10 min. Cell cultures were permeabilized with 0.2% Triton X-100 in PBS at room temperature, for 10 min. Indirect immunofluorescent and immunohistochemistry staining was performed using our previous methods [27,28]. Primary antibody against human MDA, 4-HNE, aconitase-2, 8-OHdG, HMOX-1, COX2, SOD1 and GPX1 were used. Alexa-Fluor 488 conjugated secondary antibodies was applied, and propidium iodide (PI) was used for nuclear counterstaining for immunofluorescent staining. The staining will be photographed with Zeiss laser scanning confocal microscope (LSCM510META, Thornwood, NY).

### Western blot analysis

Western blot analysis was performed using a previously reported method [29]. Equal amounts of protein measured by a BCA protein assay kit, were mixed with 6×SDS reducing sample buffer and boiled for 5 min before loading. The proteins (50μg/lane) were separated on an SDS polyacrylamide gel and transferred electronically to PVDF membranes. The membranes were blocked with 5% nonfat milk in TTBS (50 mM Tris [pH 7.5], 0.9% NaCl, and 0.1% Tween-20) for 1 h at room temperature and incubated with primary antibodies to MDA (1:200), 4-HNE (1:200), aconitase-2 (1:200), HMOX1 (1:200), COX2 (1:200), SOD1 (1:200), GPX1 (1:200), or β-actin (1:1000) overnight at 4°C. After three times washes with Tris-buffered saline with 0.05% Tween 20 for 10min each, the membranes were incubated with HRP conjugated goat anti-mouse IgG (1:2000) or goat anti-rabbit IgG (1:2000) for 1h at room temperature. The signals were detected with a chemiluminescence reagent (ECL; GE Healthcare), and the images were acquired by an imaging station (model 4000R; Eastman Kodak, Rochester, NY).

### Statistical analysis

Student’s t-test was used to compare differences between two groups. One-way ANOVA test was used to make comparisons among three or more groups, followed by Dunnett’s post-hoc test. *P* <0.05 was considered statistically significant.

## Results

### ROS production was increased in primary HCECs exposed to hyperosmotic media

**Fig 1:**
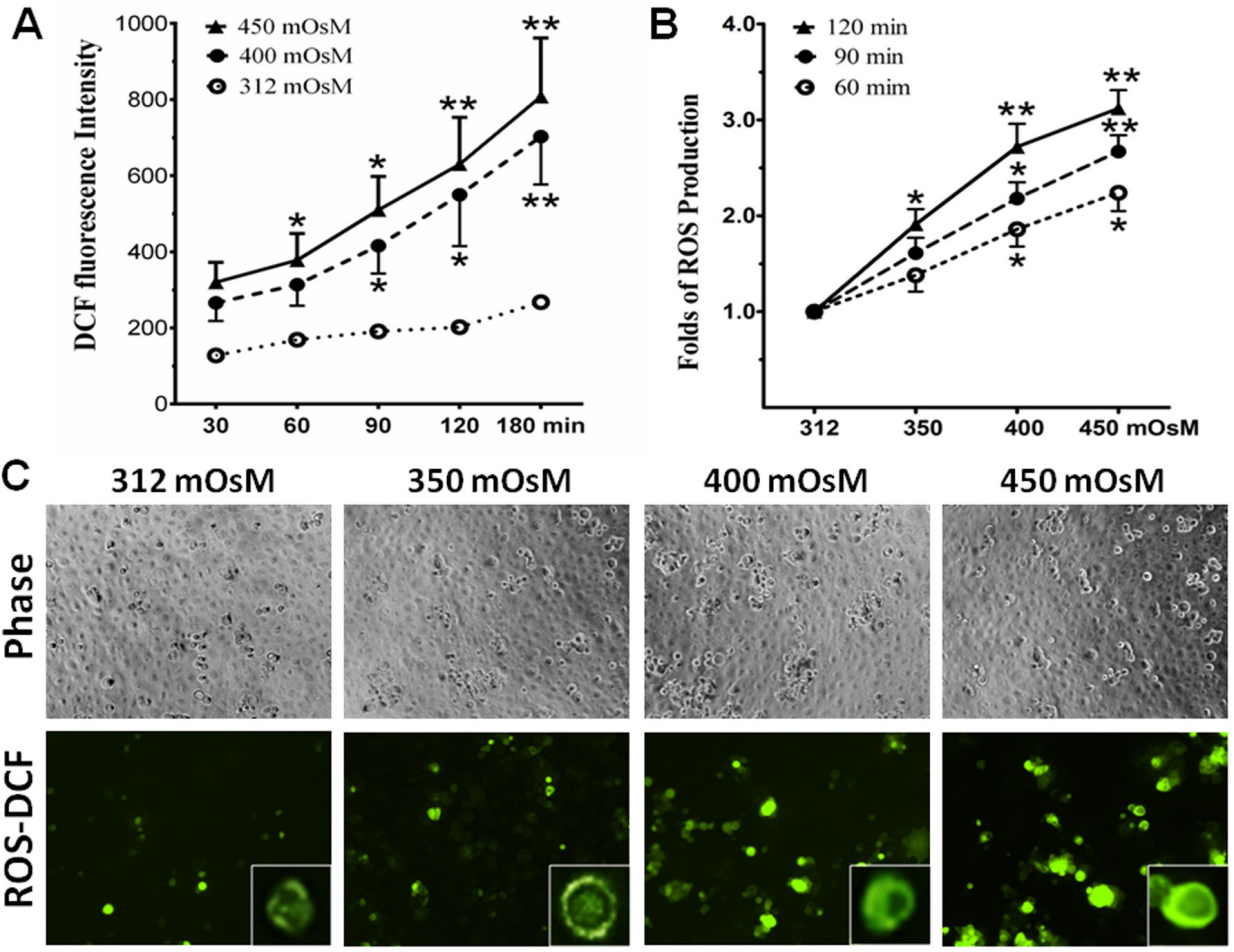
Hyperosmolarity stimulated ROS production in HCECs. **A**. Time course graph displayed a time-dependent increase of DCF fluorescence intensity in HCECs exposed to media with 400 and 450 mOsM for 30–180 min. **B.** A graph displayed osmolarity-dependent increase of relative folds of ROS production normalized by 312 mOsM control group in HCECs exposed to media with 350, 400 and 450 mOsM for 60, 90 and 120 min. Data showing Mean±SD, n = 5, * P<0.05, ** P<0.01, compared with 312 mOsM normal control. **C.** Representative images with inserts of enlarged single cell showed ROS-DCF fluorescent positive cells in HCECs exposed to media with 312–450 mOsM for 120 min; the phase images showed equal cell density in 4 groups. Magnification 100x.

Primary HCECs cultured in SHEM with iso-osmolarity (312 mOsM) were switched to hyperosmotic media from 350 to 450 mOsM for different time periods (30–180 min). The production of intracellular ROS was evaluated by the changes in DCF fluorescence intensity. The time course study revealed that ROS generation increased at time-dependent fashion in HCECs exposed to hyperosmotic media with 400 and 450 mOsM (Fig 1A). The DCF fluorescence intensity was significantly stimulated to 320±52, 378±70, 510±88, 630±123, and 808±154, respectively, when the cells exposed to 450 mOsM for 30, 60, 90, 120 and 180 min (p>0.05, p<0.05, p<0.05, p<0.01, p<0.01, n = 5, respectively). In contrast, DCF fluorescence levels were increased slightly by time in normal isosmotic group. When normalized with isosmotic controls, the relative folds of ROS production were significantly stimulated at osmolarity dependent manner, and it reached to 1.91±0.16, 2.72±0.24, and 3.12±0.19 folds, respectively, in HCECs exposed to 350, 400, and 450 mOsM (p<0.05, p<0.01, p<0.01, n = 5, respectively) for 120 min (Fig 1B). Using fluorescent microscope, the DCF fluorescence positive cells were significantly increased at osmolarity dependent manner in HCECs exposed to hypertonic media with increasing osmolarity from 312 to 350, 400, and 450 mOsM for 2 hours (Fig 1C).

### Hyperosmolarity stimulated lipid peroxidation in HCECs

**Fig 2:**
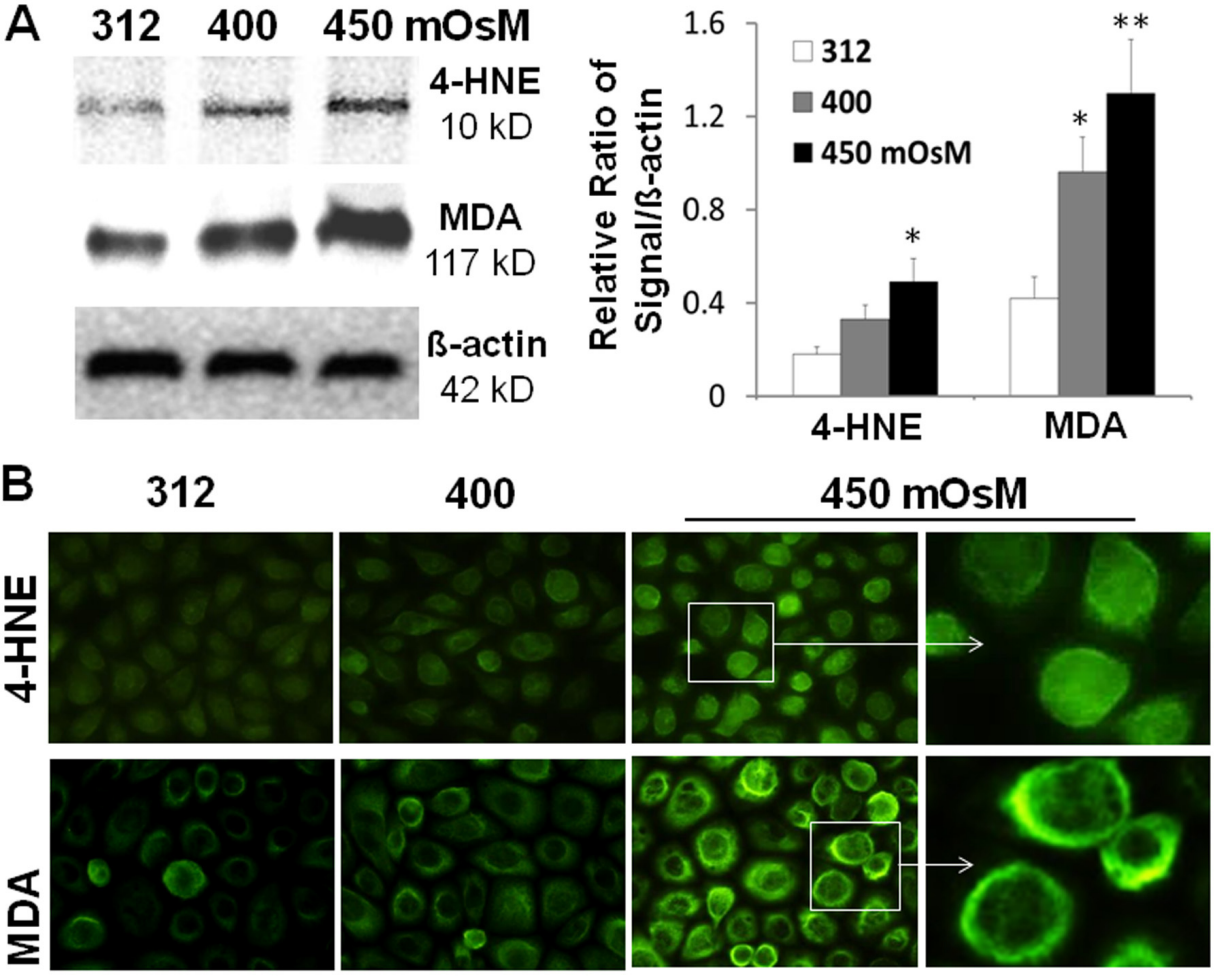
Oxidative biomarkers for cell membrane lipid peroxidation. **A.** Western blot showed increased protein levels of 4-HNE and MDA with β-actin as an internal control in primary HCECs exposed to hyperosmotic media with 400 and 450 mOsM for 24 hours. Data showing Mean±SD, n = 3, * P<0.05, ** P<0.01, compared with 312 mOsM normal control. **B.** Representative immunofluorescent images showed increased immunoreactivities of 4-HNE and MDA in HCECs exposed to different osmolarities.

Lipid peroxidation of cell membrane is one of the major consequences of ROS overproduction, which leads to the production of conjugated diene hydroperoxides and unstable substances that disintegrate into various aldehydes like HNE and MDA, the biomarkers for lipid oxidative damage. As evaluated by Western blot analysis, the protein production levels of HNE and MDA in HCECs were significantly increased at an osmolarity-dependent manner after exposure to hyperosmotic media with 400 and 450 mOsM for 24 h (Fig 2A). In contrast, the protein levels of house-keeping protein β-actin were relatively stable without changes.

Immunofluorescent staining further showed that the immunoreactivity of HNE and MDA was mainly present at cytoplasm or membrane, respectively, and their staining intensity dramatically increased in HCECs exposed to 450 mOsM (Fig 2B).

### Hyperosmolarity caused mitochondrial DNA oxidative damage in HCECs

**Fig 3:**
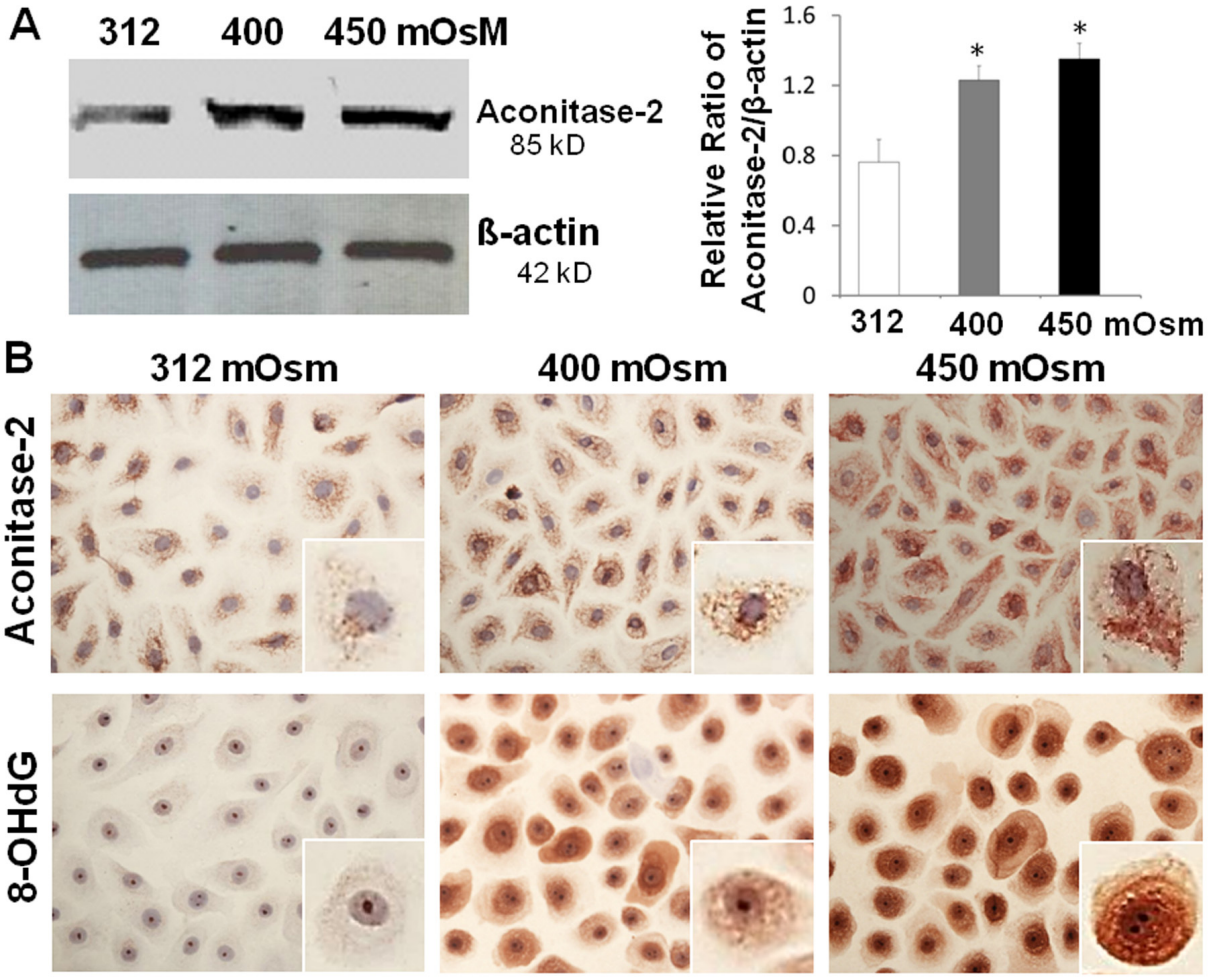
Oxidative biomarkers for mitochondrial DNA damage. **A**. Western blot showed increased aconitase-2 protein levels with β-actin as an internal control in primary HCECs exposed to hyperosmotic media with 400 and 450 mOsM for 24 hours. Data showing Mean±SD, n = 3, * P<0.05, compared with 312 mOsM normal control. **B.** Representative immunohistochemical images with insets of enlarged single cell showed increased red brown staining of aconitase-2 and 8-OHdG in HCECs exposed to different osmolarities.

In nuclear and mitochondrial DNA, 8-hydroxy-2'-deoxyguanosine (8-OHdG) is one of the predominant forms of free radical-induced oxidative lesions, and has therefore been widely used as a biomarker for oxidative stress [30]. Aconitase-2, one of the enzymes participating in the tricarboxylic acid cycle, acts as a biosensor for oxidative stress and preserves mitochondrial DNA oxidative damage [31–33]. As evaluated by Western blot analysis, aconitase-2 protein was found to increase markedly at an osmolarity-dependent manner in HCECs exposed to hyperosmotic media with 400 and 450 mOsM for 24 hours when compared to that in isosmolar medium, as well as house-keeping protein β-actin (Fig 3A). The immunohistochemical staining (Fig 3B) further showed that the punctate staining of aconitase-2 in cytoplasm, representing the mitochondria; and the density and intensity of the punctate staining of aconitase-2 were markedly increased in the cells exposed to hypertonic media at 400–450 mOsM when compared with cells in isosmolar medium. Interestingly, the markedly increased red brown staining of an oxidized DNA product 8-OHdG was also observed to be localized in cytoplasm or nuclear compartment of HCECs exposed to hyperosmotic media (400–450 mOsM) for 24 hours while the most cells were stained negative in isosmolar media (Fig 3B). Furthermore, the enlarged single cell images of 8-OHdG staining showed its homogenous staining in nuclear, while a punctate pattern in cytoplasm. The density and intensity of 8-OHdG punctate staining in cytoplasm were markedly increased in the cells exposed to hypertonic media at 400 and 450 mOsM, indicating mitochondrial DNA damage.

### Oxygenases HMOX1 and COX2 were induced in HCECs exposed to hyperosmotic media

**Fig 4:**
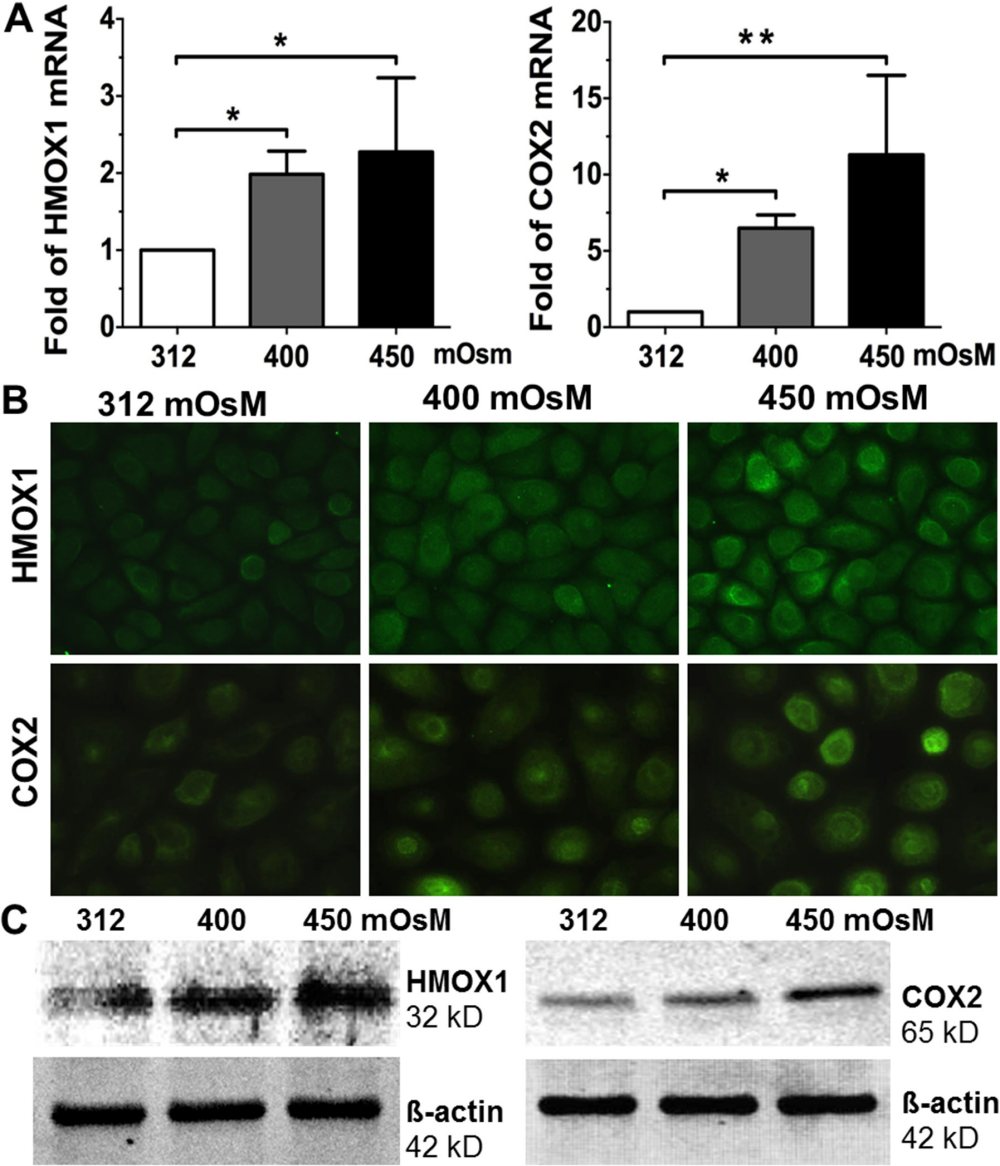
The increased mRNA and protein levels of oxygenases HMOX1 and COX2 induced by hyperosmotic media in HCECs. Primary HCECs exposed to media at 312, 400 and 450 mOsM for 4 hours were lysed for mRNA expression by RT-qPCR (**A**), and the cells treated for 24 hours were performed for immunofluorescent staining (**B**) or lysed in RIPA buffer for Western blotting (**C**), to determine the mRNA expression and protein production of HMOX1 and COX2. Data showing mean±SD, n = 5, * P<0.05, ** P<0.01, compared with 312 mOsM normal control.

HMOX1, a 32 kD protein, is an inducible isoform in response to stress such as oxidative stress, hypoxia, heavy metals and cytokines [34,35]. As prostaglandin-endoperoxide synthase COX2 is an enzyme that mediates oxidative stress [36,37]. As shown in Fig 4A, HMOX1 mRNA expression significantly increased to 1.99±0.60, and 2.27±0.96 fold, respectively, in HCECs exposed to increasing hyperosmolarity, 400 and 450 mOsM (p<0.05, p<0.05, n = 5, respectively) for 4 hours when compared with the isosmotic group, as evaluated by RT-qPCR. COX2 mRNA levels also dramatically increased to 6.48±1.73, and 11.29±6.29 fold, respectively, in HCECs when switch from normal medium to increasing hyperosmolarity, 400 or 450 mOsM (p<0.01, p<0.01, n = 5, respectively) for 4 hours. Hyperosmolarity induced HMOX1 and COX2 were confirmed at protein levels as determined by immunofluorescent staining (Fig 4B). The immunoreactivity of HMOX1 and COX2 were located mainly in the cytoplasm of primary HCECs. The intensity of HMOX1 and COX2 immunoreactivity was significantly induced in an osmolarity-dependent manner in HCECs exposed to media with increasing hyperosmolarity (400 and 450 mOsM) for 24 hours. This pattern of response to hyperosmolarity by HCECs was further confirmed by Western blot analysis, which showed that these two oxygenases, 32 kD HMOX1 and 65 kD COX2, were indeed induced osmolarity-dependently by increasing hyperosmolarity in HCECs (Fig 4C).

### Production of anti-oxidative enzymes SOD1 and GPX1 was suppressed by hyperosmolarity

**Fig 5:**
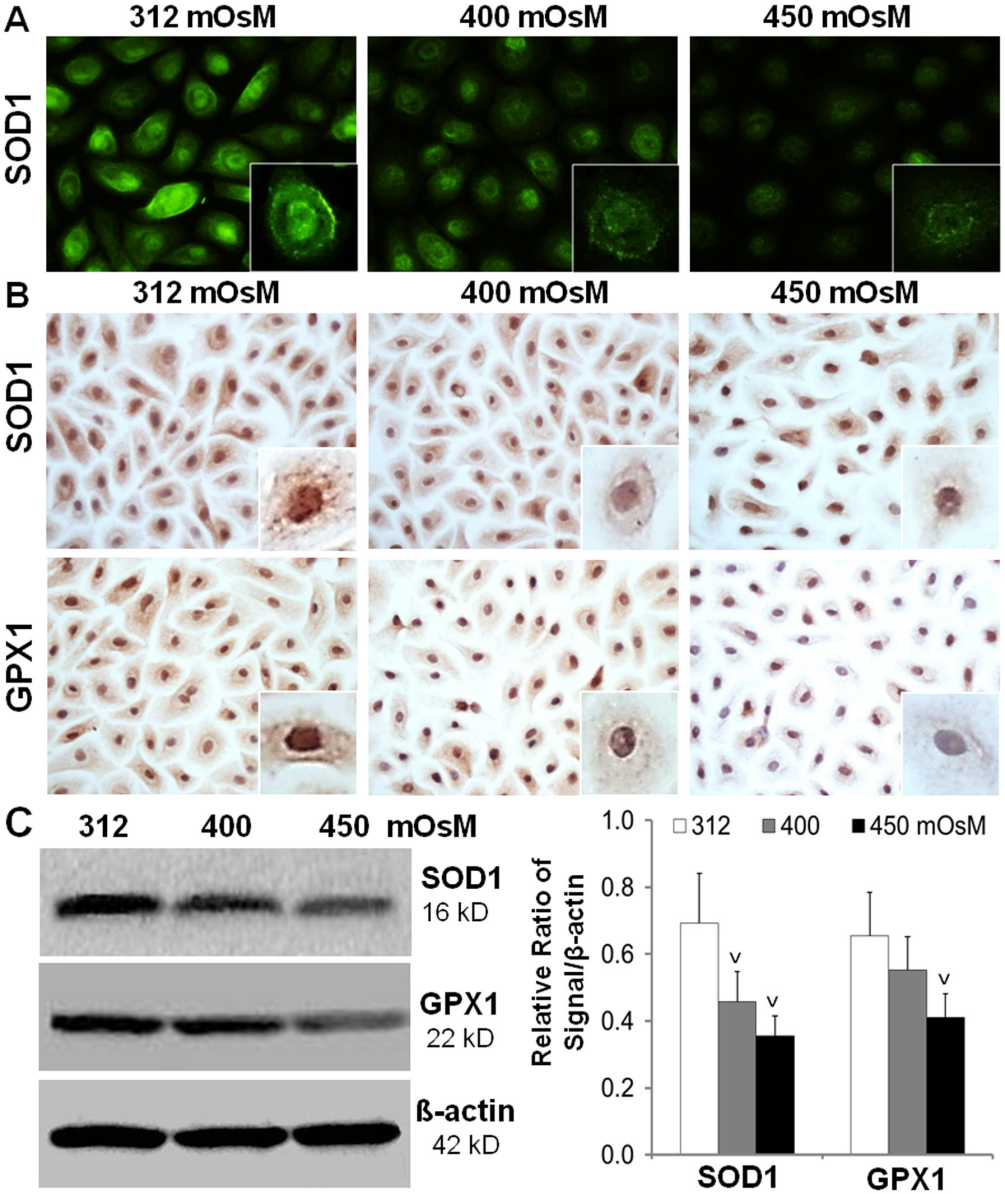
The reduced production of anti-oxidative enzymes SOD1 and GPX1 in HCECs exposed to hyperosmotic stress. Primary HCECs exposed to media at 312, 400 and 450 mOsM for 24 hours were performed for immunofluorescent staining (**A**), or immunohistochemical staining (**B**), or lysed in RIPA buffer for Western blotting (**C**), to determine the protein production of SOD1 and GPX1. Data showing mean±SD, n = 4, ^V^ P<0.05, ^VV^ P<0.01, compared with 312 mOsM normal control.

Anti-oxidative enzymes are suggested as protective markers of oxidative stress. For examples, SOD, which eliminates O_2_^-^ to produce H_2_O_2_, can scavenge the toxic effect of ROS; and H_2_O_2_ is eliminated by GPX and catalase [38]. Oxidative stress has been speculated to cause antioxidant consumption, which results in a decline of antioxidant levels [39]. As shown in Fig 5A, the immunoreactivity of SOD1 (Cu-Zn-SOD) was strongly localized in cytoplasm and nucleus of HCECs cultured in normal isosmotic medium. However, SOD1 protein reactivity was largely suppressed in HCECs cultured in media with increasing hyperosmolarity (400 and 450 mOsM) as evaluated by both immunofluorescent and immunohistochemical staining (Fig 5A & 5B). GPX1 protein was also observed to be located at cytoplasm and nucleus of HCECs in isosmotic medium although not as strong as to SOD1. The red-brown staining of GPX1 was significantly suppressed in HCECs exposed to increasing hyperosmolarity (400–450 mOsM) (Fig 5B). The pattern of anti-oxidative enzymes, 16 kD SOD1 and 22 kD GPX1, osmolarity-dependently suppressed by increasing hyperosmolarity in HCECs was further confirmed by Western blot analysis with β-actin as internal control (Fig 5C).

## Discussion

Previous studies including ours have shown the increased expression and production of proinflammatory cytokines (TNF-α, IL-1β, IL-6), chemokines (IL-8), and matrix metalloproteinases (MMP-13, -3, -9) in primary cultured HCECs exposed to hyperosmotic media [18,29,40,41]. However, few studies have shown biomarkers of oxidative damage of corneal epithelial cells induced by hyperosmolarity. In this study, we investigated the ROS production, major cellular biomarkers of oxidative damage, and imbalanced oxidative and anti-oxidative enzymes in primary HCECs exposed to hyperosmotic media, an established in vitro culture model for hyperosmolarity-induced dry eye.

### ROS overproduction was induced by hyperosmolarity in HCECs

Cells generate energy by reducing molecular oxygen to water. During this process, small amounts of partially reduced reactive oxygen molecules are produced as an unavoidable byproduct of mitochondrial respiration. Some of these molecules are free radicals referred to as ROS that can cause cell oxidative injury [38,42]. Previous studies have shown that ROS production was increased as early as 15 min in response to hyperosmolarity in cultured mouse renal inner medullary cells exposed to NaCl or Urea at 600 mOsM [43], or cultured collecting duct cells in medium with 550 mOsM [44]. In this study, we observed a similar response pattern of ROS in cultured HCECs exposed to media with increased osmolarity (350–450mOsM). The ROS production increased as early as 30 min, reached significant levels at 60 min, and ROS levels were stimulated by increasing osmolarity at the time- (30–180 min) and osmolarity-dependent (350–450 mOsM) manner as determined by DCF fluorescent intensity and the number of DCF fluorescence positive cells (Fig 1).

### Oxidative markers for lipid peroxidation and mitochondrial DNA damage induced by hyperosmolarity in HCECs

Over-produced ROS results in oxidative stress while cells are able to overcome mild oxidative stress and regain its original state. Intense and severe oxidative stress may trigger apoptosis, and even cause necrosis and finally cell death [45]. There are at least three reactions relevant to oxidative damage: lipid peroxidation of membranes, oxidative modification of proteins, and oxidative damage to DNA [38]. Herein, we investigated the oxidative biomarkers that judge the cell damage conditions by hyperosmolarity.

ROS free radicals directly oxidize various macromolecules, including lipids. MDA and 4-HNE are major end-products of oxidation of polyunsaturated fatty acids, and are frequently measured as indicators of lipid peroxidation and oxidative stress. Lipid peroxides and their breakdown products can directly or indirectly affect many functions integral to cellular and organ homeostasis. For example, MDA is a highly toxic molecule, and its interaction with DNA and proteins has been referred to as potentially mutagenic and atherogenic [5,46]. HNE is a toxic messenger of oxygen free radicals and undergoes reactions with proteins, peptides, phospholipids and nucleic acids, which has cytotoxic, mutagenic and genotoxic signal effects. They both have been identified as the most cytotoxic breakdown products generated from lipid peroxidation [47–49]. The levels of HNE in tear and conjunctiva were observed to be significantly higher in Sjögren syndrome (SS) compared to normal controls [21,50]. In present study, we showed that hyperosmolarity significantly stimulated the production of MDA and HNE at osmolarity-dependent manner in HCECs as evaluated by Western blotting and immunofluorescent staining (Fig 2).

Mitochondria as "cellular power plants” are a source of chemical energy. In addition to supplying cellular energy, mitochondria are involved in other tasks such as signaling, cellular differentiation, cell death, as well as the control of the cell cycle and cell growth. Oxidative stress of mitochondria has been known to associate with many pathologic processes influencing cell death and survival such as apoptosis, autophagy, and plasticity. Aconitase-2, a multifunctional enzyme, possesses an iron-sulfur cluster that plays a role in the electron-transfer reactions of oxidative phosphorylation [51], acts as a sensor in the redox regulation of metabolism by O_2._ Aconitase-2 increases with high mitochondrial activity in the cells under oxidative stress [52,53]. 8-OHdG is a product of oxidative DNA damage following specific enzymatic cleavage by ROS-induced 8-hydroxylation of the guanine base in mitochondrial and nuclear DNA [54]. The repair of damaged DNA triggers the excretion of 8-OHdG in the cell medium without further metabolism [55]. Therefore, aconitase-2 activity and the released 8-OHdG have been widely used as sensitive and reliable makers of the oxidative mitochondria DNA damage [56]. Our results showed that the production of both aconitase-2 and 8-OHdG increased in HCECs exposed to media with increasing osmolarity from 312 mOsM to 400 and 450 mOsM, as evaluated by Western blot analysis and immunohistochemical staining (Fig 3). The results suggest that potential mitochondria DNA damage may occurred in HCECs under hyperosmolarity condition.

### Hyperosmolarity disrupted the balance of oxygenases and anti-oxidative enzymes in HCECs

HMOX1 and COX2 are two major oxygenases highly induced by a variety of agents causing oxidative stress. HMOX1 is an inducible isoform in response to stress such as oxidative stress, hypoxia, heavy metals, cytokines, and was considered as an important protectant in response to oxidative stress in human eye [57–59]. COX, contains two major isoforms COX1 and COX2, is the enzyme that catalyzes the metabolism of arachidonic acid to prostaglandins. Upregulation of COX2 is a common feature of inflammation by oxidative stress. COX2 is reported to be stimulated by lipid peroxidation, and to mediate oxidative DNA damage [23,60,61]. However, these oxygenases have not been well investigated in dry eye conditions. In this study, we observed that the mRNA expression and protein production of HMOX1 and COX2 were markedly induced at osmolarity-dependent manner by hyperosmolarity in HCECs, as evaluated by RT-qPCR, immunofluorescent staining and Western blot analysis (Fig 4).

Antioxidant enzymes form the first line of defense in organisms against free radicals and toxic reactants by metabolizing them to innocuous byproducts [62]. SOD, made up of three members, Cu-Zn-SOD (SOD1), Mn-SOD (SOD2) and extracelluar SOD (SOD3), is one of the main antioxidant systems that catalyze the dismutation of superoxide into oxygen and hydrogen peroxide [63]. GPX is an enzyme family with peroxidase activity whose main biological role is to protect the organism from oxidative damage by reducing lipid hydroperoxides to their corresponding alcohols and to reduce free hydrogen peroxide to water [64,65]. The activities of SOD isoenzymes have been observed in tears, cornea, and the other part of the human eye [63]. GPX1 was found to be expressed in corneal and lens epithelia, and retina in human adult eye, and inhibit cellular inflammatory responses [65]. However, few studies have been investigated on the role of these antioxidant enzymes in dry eye disease so far.

Our findings shown in Fig 5 revealed that hyperosmolarity interrupted the antioxidant defense system by reducing the production of antioxidant enzymes, SOD1 and GPX1, at osmolarity-dependent manner in HCECs exposed to hyperosmotic media, as evaluated by immunofluorescent and immunohistochemical staining and Western blot analysis. These data suggest that the oxidative damage may be caused by imbalance between the increased oxygenases HMOX1 and COX2 and suppressed antioxidant enzymes SOD1 and GPX1 in HCECs exposed to hyperosmotic media.

Oxidative stress and damage have been recognized to involve in a variety of inflammatory diseases, including ocular surface disease and dry eye syndromes. Ocular surface lipid oxidative stress status was found to be strongly correlated to marked tear instability, ocular surface epithelial damage, and increased inflammatory infiltrates in patients with atopic keratoconjunctivitis [66]. The mitochondria-induced oxidative damage has a strong relationship with lacrimal gland inflammation and dysfunction, resulting in dry eye disease in a conditional transgenic mouse model [42]. Increase of the oxidative stress status in the conjunctiva of SS patients appears to have a role in the pathogenesis of dry eye disease. A close relationship among ROS production, lipid peroxidation related membrane damage, and inflammatory processes was observed in dry eye patients with Sjögren syndrome [21,67]. Our findings in the present studies provide strong evidence that oxidative stress may play an important role in inflammatory dry eye disease.

In conclusion, our comprehensive findings demonstrate that hyperosmolarity induces oxidative stress in HCECs by stimulating ROS generation, increasing the expression and production of oxygenases HMOX1 and COX2, and suppressing the protein levels of antioxidant enzymes SOD1 and GPX1, which in turns caused significantly increased biomarkers of cellular oxidative damage in lipid peroxidation of cell membranes (HNE and MDA) and in mitochondrial DNA (8-OHdG and aconitase-2). Further investigation is necessary and will be more interesting to explore the protective effects and mechanism of anti-oxidants on dry eye using in vitro, ex-vivo and/or in vivo dry eye models, which will further cover extracellular ROS production by dry eye conditions, ROS elimination and oxidative stress protection by anti-oxidative enzymes and other agents.

## References

[1] Devasagayam TP, Tilak JC, Boloor KK, Sane KS, Ghaskadbi SS, Lele RD. Free radicals and antioxidants in human health: current status and future prospects. J Assoc Physicians India. 2004;52: 794–804. 15909857

[2] Maynard S, Keijzers G, Gram M, Desler C, Bendix L, Budtz-Jorgensen E, et al Relationships between human vitality and mitochondrial respiratory parameters, reactive oxygen species production and dNTP levels in peripheral blood mononuclear cells. Aging (Albany NY). 2013;5: 850–864. 24304678 10.18632/aging.100618 PMC3868727

[3] Van HB, Woshner V, Santos JH. Role of mitochondrial DNA in toxic responses to oxidative stress. DNA Repair (Amst). 2006;5: 145–152. 15878696 10.1016/j.dnarep.2005.03.002

[4] Figueira TR, Barros MH, Camargo AA, Castilho RF, Ferreira JC, Kowaltowski AJ, et al Mitochondria as a source of reactive oxygen and nitrogen species: from molecular mechanisms to human health. Antioxid Redox Signal. 2013;18: 2029–2074. 10.1089/ars.2012.4729 23244576

[5] Waldeck AR, Stocker R. Radical-initiated lipid peroxidation in low density lipoproteins: insights obtained from kinetic modeling. Chem Res Toxicol. 1996;9: 954–964. 8870982 10.1021/tx960057s

[6] Brambilla G, Sciaba L, Faggin P, Maura A, Marinari UM, Ferro M, et al Cytotoxicity, DNA fragmentation and sister-chromatid exchange in Chinese hamster ovary cells exposed to the lipid peroxidation product 4-hydroxynonenal and homologous aldehydes. Mutat Res. 1986;171: 169–176. 3755798 10.1016/0165-1218(86)90051-0

[7] Esterbauer H, Schaur RJ, Zollner H. Chemistry and biochemistry of 4-hydroxynonenal, malonaldehyde and related aldehydes. Free Radic Biol Med. 1991;11: 81–128. 1937131 10.1016/0891-5849(91)90192-6

[8] Sosa V, Moline T, Somoza R, Paciucci R, Kondoh H, LLeonart ME. Oxidative stress and cancer: an overview. Ageing Res Rev. 2013;12: 376–390. 10.1016/j.arr.2012.10.004 23123177

[9] Delanty N, Dichter MA. Oxidative injury in the nervous system. Acta Neurol Scand. 1998;98: 145–153. 9786609 10.1111/j.1600-0404.1998.tb07285.x

[10] Ward PA. Oxidative stress: acute and progressive lung injury. Ann N Y Acad Sci. 2010;1203: 53–59. 10.1111/j.1749-6632.2010.05552.x 20716283

[11] Shah AA, Sinha AA. Oxidative stress and autoimmune skin disease. Eur J Dermatol. 2013;23: 5–13. 10.1684/ejd.2012.1884 23420016

[12] Pescosolido N, Imperatrice B, Karavitis P. The aging eye and the role of L-carnitine and its derivatives. Drugs R D. 2008;9 Suppl 1: 3–14. 10.2165/0126839-200809001-00002 19105587

[13] Uchino Y, Kawakita T, Miyazawa M, Ishii T, Onouchi H, Yasuda K, et al Oxidative stress induced inflammation initiates functional decline of tear production. PLoS One. 2012;7: e45805 10.1371/journal.pone.0045805 23071526 PMC3465290

[14] Zheng Q, Ren Y, Reinach PS, She Y, Xiao B, Hua S, et al Reactive oxygen species activated NLRP3 inflammasomes prime environment-induced murine dry eye. Exp Eye Res. 2014;125: 1–8. 10.1016/j.exer.2014.05.001 24836981

[15] Baudouin C, Aragona P, Messmer EM, Tomlinson A, Calonge M, Boboridis KG, et al Role of hyperosmolarity in the pathogenesis and management of dry eye disease: proceedings of the OCEAN group meeting. Ocul Surf. 2013;11: 246–258. 10.1016/j.jtos.2013.07.003 24112228

[16] Lemp MA, Bron AJ, Baudouin C, Benitez del Castillo JM, Geffen D, Tauber J, et al Tear osmolarity in the diagnosis and management of dry eye disease. Am J Ophthalmol. 2011;151: 792–798. 10.1016/j.ajo.2010.10.032 21310379

[17] Julio G, Lluch S, Pujol P, Merindano MD. Effects of tear hyperosmolarity on conjunctival cells in mild to moderate dry eye. Ophthalmic Physiol Opt. 2012;32: 317–323. 10.1111/j.1475-1313.2012.00915.x 22620852

[18] Li D-Q, Chen Z, Song XJ, Luo L, Pflugfelder SC. Stimulation of matrix metalloproteinases by hyperosmolarity via a JNK pathway in human corneal epithelial cells. Invest Ophthalmol Vis Sci. 2004;45: 4302–4311. 15557436 10.1167/iovs.04-0299

[19] Luo L, Li D-Q, Doshi A, Farley W, Corrales RM, Pflugfelder SC. Experimental dry eye stimulates production of inflammatory cytokines and MMP-9 and activates MAPK signaling pathways on the ocular surface. Invest Ophthalmol Vis Sci. 2004;45: 4293–4301. 15557435 10.1167/iovs.03-1145

[20] Luo L, Li D-Q, Corrales RM, Pflugfelder SC. Hyperosmolar saline is a proinflammatory stress on the mouse ocular surface. Eye Contact Lens. 2005;31: 186–193. 16163009 10.1097/01.icl.0000162759.79740.46

[21] Wakamatsu TH, Dogru M, Matsumoto Y, Kojima T, Kaido M, Ibrahim OM, et al Evaluation of lipid oxidative stress status in Sjogren syndrome patients. Invest Ophthalmol Vis Sci. 2013;54: 201–210. 10.1167/iovs.12-10325 23150615

[22] Nakamura S, Shibuya M, Nakashima H, Hisamura R, Masuda N, Imagawa T, et al Involvement of oxidative stress on corneal epithelial alterations in a blink-suppressed dry eye. Invest Ophthalmol Vis Sci. 2007;48: 1552–1558. 17389484 10.1167/iovs.06-1027

[23] Higuchi A, Inoue H, Kawakita T, Ogishima T, Tsubota K. Selenium compound protects corneal epithelium against oxidative stress. PLoS One. 2012;7: e45612 10.1371/journal.pone.0045612 23049824 PMC3458096

[24] Kim HS, Jun Song X, de Paiva CS, Chen Z, Pflugfelder SC, Li D-Q. Phenotypic characterization of human corneal epithelial cells expanded ex vivo from limbal explant and single cell cultures. Exp Eye Res. 2004;79: 41–49. 15183099 10.1016/j.exer.2004.02.015 PMC2906376

[25] Yoon KC, de Paiva CS, Qi H, Chen Z, Farley WJ, Li DQ, et al Expression of Th-1 chemokines and chemokine receptors on the ocular surface of C57BL/6 mice: effects of desiccating stress. Invest Ophthalmol Vis Sci. 2007;48: 2561–2569. 17525185 10.1167/iovs.07-0002

[26] Ma P, Bian F, Wang Z, Zheng X, Chotikavanich S, Pflugfelder SC, et al Human corneal epithelium-derived thymic stromal lymphopoietin links the innate and adaptive immune responses via TLRs and Th2 cytokines. Invest Ophthalmol Vis Sci. 2009;50: 2702–2709. 10.1167/iovs.08-3074 19151401 PMC5496816

[27] Chen Z, de Paiva CS, Luo L, Kretzer FL, Pflugfelder SC, Li D-Q. Characterization of putative stem cell phenotype in human limbal epithelia. Stem Cells. 2004;22: 355–366. 15153612 10.1634/stemcells.22-3-355 PMC2906385

[28] Li D-Q, Tseng SC. Three patterns of cytokine expression potentially involved in epithelial-fibroblast interactions of human ocular surface. J Cell Physiol. 1995;163: 61–79. 7896901 10.1002/jcp.1041630108

[29] Li D-Q, Luo L, Chen Z, Kim HS, Song XJ, Pflugfelder SC. JNK and ERK MAP kinases mediate induction of IL-1beta, TNF-alpha and IL-8 following hyperosmolar stress in human limbal epithelial cells. Exp Eye Res. 2006;82: 588–596. 16202406 10.1016/j.exer.2005.08.019 PMC2198933

[30] Valavanidis A, Vlachogianni T, Fiotakis C. 8-hydroxy-2'-deoxyguanosine (8-OHdG): A critical biomarker of oxidative stress and carcinogenesis. J Environ Sci Health C Environ Carcinog Ecotoxicol Rev. 2009;27: 120–139. 10.1080/10590500902885684 19412858

[31] Kim SJ, Cheresh P, Williams D, Cheng Y, Ridge K, Schumacker PT, et al Mitochondria-targeted Ogg1 and aconitase-2 prevent oxidant-induced mitochondrial DNA damage in alveolar epithelial cells. J Biol Chem. 2014;289: 6165–6176. 10.1074/jbc.M113.515130 24429287 PMC3937682

[32] Raukas M, Rebane R, Mahlapuu R, Jefremov V, Zilmer K, Karelson E, et al Mitochondrial oxidative stress index, activity of redox-sensitive aconitase and effects of endogenous anti- and pro-oxidants on its activity in control, Alzheimer's disease and Swedish Familial Alzheimer's disease brain. Free Radic Res. 2012;46: 1490–1495. 10.3109/10715762.2012.728286 22962855

[33] Chen XJ, Wang X, Kaufman BA, Butow RA. Aconitase couples metabolic regulation to mitochondrial DNA maintenance. Science. 2005;307: 714–717. 15692048 10.1126/science.1106391

[34] Tyrrell R. Redox regulation and oxidant activation of heme oxygenase-1. Free Radic Res. 1999;31: 335–340. 10517538 10.1080/10715769900300901

[35] Boyle JJ. Heme and haemoglobin direct macrophage Mhem phenotype and counter foam cell formation in areas of intraplaque haemorrhage. Curr Opin Lipidol. 2012;23: 453–461. 10.1097/MOL.0b013e328356b145 22777293

[36] Madrigal JL, Garcia-Bueno B, Caso JR, Perez-Nievas BG, Leza JC. Stress-induced oxidative changes in brain. CNS Neurol Disord Drug Targets. 2006;5: 561–568. 17073658 10.2174/187152706778559327

[37] Kleiveland CR, Kassem M, Lea T. Human mesenchymal stem cell proliferation is regulated by PGE2 through differential activation of cAMP-dependent protein kinase isoforms. Exp Cell Res. 2008;314: 1831–1838. 10.1016/j.yexcr.2008.02.004 18395713

[38] Wakamatsu TH, Dogru M, Tsubota K. Tearful relations: oxidative stress, inflammation and eye diseases. Arq Bras Oftalmol. 2008;71: 72–79. 19274416 10.1590/s0004-27492008000700015

[39] Polidori MC, Stahl W, Eichler O, Niestroj I, Sies H. Profiles of antioxidants in human plasma. Free Radic Biol Med. 2001;30: 456–462. 11182517 10.1016/s0891-5849(00)00345-2

[40] Pan Z, Wang Z, Yang H, Zhang F, Reinach PS. TRPV1 activation is required for hypertonicity-stimulated inflammatory cytokine release in human corneal epithelial cells. Invest Ophthalmol Vis Sci. 2011;52: 485–493. 10.1167/iovs.10-5801 20739465 PMC3053292

[41] Png E, Samivelu GK, Yeo SH, Chew J, Chaurasia SS, Tong L. Hyperosmolarity-mediated mitochondrial dysfunction requires Transglutaminase-2 in human corneal epithelial cells. J Cell Physiol. 2011;226: 693–699. 10.1002/jcp.22389 20717931

[42] Uchino Y, Kawakita T, Ishii T, Ishii N, Tsubota K. A new mouse model of dry eye disease: oxidative stress affects functional decline in the lacrimal gland. Cornea. 2012;31 Suppl 1: S63–S67. 10.1097/ICO.0b013e31826a5de1 23038038

[43] Zhang Z, Dmitrieva NI, Park JH, Levine RL, Burg MB. High urea and NaCl carbonylate proteins in renal cells in culture and in vivo, and high urea causes 8-oxoguanine lesions in their DNA. Proc Natl Acad Sci U S A. 2004;101: 9491–9496. 15190183 10.1073/pnas.0402961101 PMC439004

[44] Yang T, Zhang A, Honeggar M, Kohan DE, Mizel D, Sanders K, et al Hypertonic induction of COX-2 in collecting duct cells by reactive oxygen species of mitochondrial origin. J Biol Chem. 2005;280: 34966–34973. 16024921 10.1074/jbc.M502430200

[45] Valko M, Morris H, Cronin MT. Metals, toxicity and oxidative stress. Curr Med Chem. 2005;12: 1161–1208. 15892631 10.2174/0929867053764635

[46] Del RD, Stewart AJ, Pellegrini N. A review of recent studies on malondialdehyde as toxic molecule and biological marker of oxidative stress. Nutr Metab Cardiovasc Dis. 2005;15: 316–328. 16054557 10.1016/j.numecd.2005.05.003

[47] Esterbauer H, Schaur RJ, Zollner H. Chemistry and biochemistry of 4-hydroxynonenal, malonaldehyde and related aldehydes. Free Radic Biol Med. 1991;11: 81–128. 1937131 10.1016/0891-5849(91)90192-6

[48] Eckl PM, Ortner A, Esterbauer H. Genotoxic properties of 4-hydroxyalkenals and analogous aldehydes. Mutat Res. 1993;290: 183–192. 7694109 10.1016/0027-5107(93)90158-c

[49] Uchida K. 4-Hydroxy-2-nonenal: a product and mediator of oxidative stress. Prog Lipid Res. 2003;42: 318–343. 12689622 10.1016/s0163-7827(03)00014-6

[50] Andrade AS, Salomon TB, Behling CS, Mahl CD, Hackenhaar FS, Putti J, et al Alpha-lipoic acid restores tear production in an animal model of dry eye. Exp Eye Res. 2014;120: 1–9. 10.1016/j.exer.2013.12.014 24394592

[51] Berg F, Gustafson U, Andersson L. The uncoupling protein 1 gene (UCP1) is disrupted in the pig lineage: a genetic explanation for poor thermoregulation in piglets. PLoS Genet. 2006;2: e129 16933999 10.1371/journal.pgen.0020129 PMC1550502

[52] Armstrong JS, Whiteman M, Yang H, Jones DP. The redox regulation of intermediary metabolism by a superoxide-aconitase rheostat. BioEssays. 2004;26: 894–900. 15273991 10.1002/bies.20071

[53] Chen YH, Chiang YH, Ma HI. Analysis of spatial and temporal protein expression in the cerebral cortex after ischemia-reperfusion injury. J Clin Neurol. 2014;10: 84–93. 10.3988/jcn.2014.10.2.84 24829593 PMC4017024

[54] Marczynski B, Rozynek P, Kraus T, Schlosser S, Raithel HJ, Baur X. Levels of 8-hydroxy-2'-deoxyguanosine in DNA of white blood cells from workers highly exposed to asbestos in Germany. Mutat Res. 2000;468: 195–202. 10882896 10.1016/s1383-5718(00)00053-x

[55] Dong QY, Cui Y, Chen L, Song J, Sun L. Urinary 8-hydroxydeoxyguanosine levels in diabetic retinopathy patients. Eur J Ophthalmol. 2008;18: 94–98. 18203092 10.1177/112067210801800116

[56] Patel PR, Bevan RJ, Mistry N, Lunec J. Evidence of oligonucleotides containing 8-hydroxy-2'-deoxyguanosine in human urine. Free Radic Biol Med. 2007;42: 552–558. 17275687 10.1016/j.freeradbiomed.2006.11.025

[57] Woo JM, Shin DY, Lee SJ, Joe Y, Zheng M, Yim JH, et al Curcumin protects retinal pigment epithelial cells against oxidative stress via induction of heme oxygenase-1 expression and reduction of reactive oxygen. Mol Vis. 2012;18: 901–908. 22539869 PMC3335783

[58] Cano M, Thimmalappula R, Fujihara M, Nagai N, Sporn M, Wang AL, et al Cigarette smoking, oxidative stress, the anti-oxidant response through Nrf2 signaling, and Age-related Macular Degeneration. Vision Res. 2010;50: 652–664. 10.1016/j.visres.2009.08.018 19703486 PMC3575185

[59] Gronert K. Resolution, the grail for healthy ocular inflammation. Exp Eye Res. 2010;91: 478–485. 10.1016/j.exer.2010.07.004 20637194 PMC2939166

[60] Bao F, Chen Y, Dekaban GA, Weaver LC. An anti-CD11d integrin antibody reduces cyclooxygenase-2 expression and protein and DNA oxidation after spinal cord injury in rats. J Neurochem. 2004;90: 1194–1204. 15312174 10.1111/j.1471-4159.2004.02580.x

[61] Lee SH, Williams MV, Dubois RN, Blair IA. Cyclooxygenase-2-mediated DNA damage. J Biol Chem. 2005;280: 28337–28346. 15964853 10.1074/jbc.M504178200

[62] Valentine JS, Doucette PA, Zittin PS. Copper-zinc superoxide dismutase and amyotrophic lateral sclerosis. Annu Rev Biochem. 2005;74: 563–593. 15952898 10.1146/annurev.biochem.72.121801.161647

[63] Behndig A, Svensson B, Marklund SL, Karlsson K. Superoxide dismutase isoenzymes in the human eye. Invest Ophthalmol Vis Sci. 1998;39: 471–475. 9501855

[64] Zhang Y, Zhang L, Sun D, Li Z, Wang L, Liu P. Genetic polymorphisms of superoxide dismutases, catalase, and glutathione peroxidase in age-related cataract. Mol Vis. 2011;17: 2325–2332. 21921984 PMC3171498

[65] Chang YC, Chang WC, Hung KH, Yang DM, Cheng YH, Liao YW, et al The generation of induced pluripotent stem cells for macular degeneration as a drug screening platform: identification of curcumin as a protective agent for retinal pigment epithelial cells against oxidative stress. Front Aging Neurosci. 2014;6: 191 10.3389/fnagi.2014.00191 25136316 PMC4117985

[66] Wakamatsu TH, Dogru M, Ayako I, Takano Y, Matsumoto Y, Ibrahim OM, et al Evaluation of lipid oxidative stress status and inflammation in atopic ocular surface disease. Mol Vis. 2010;16: 2465–2475. 21139696 PMC2994734

[67] Cejkova J, Ardan T, Cejka C, Malec J, Jirsova K, Filipec M, et al Ocular surface injuries in autoimmune dry eye. The severity of microscopical disturbances goes parallel with the severity of symptoms of dryness. Histol Histopathol. 2009;24: 1357–1365. 19688700 10.14670/HH-24.1357

